# Supplementary material for: School Burnout after COVID-19, Prevalence and Role of Different Risk and Protective Factors in Preteen Students
Source: Children (Basel). 2023 Apr 30;10(5):823. doi: 10.3390/children10050823 (PMC10217600; doi:10.3390/children10050823)
Supplement: Supplementary file 1 [file children-10-00823-s001.zip › children-2341670-supplementary.pdf]

Table S1: Results of correlations between burnout and risk factors

	BO	EE	CYN	INAD
**Individual factors**				
**Somatic symptoms**				
Lack of energy	.541**	.494**	.521**	.425**
Pain (stomach, head, back)	.493**	.446**	.432**	.446**
Fatigue	.587**	.554**	.548**	.455**
Difficulty sleeping	.454**	.455**	.402**	.342**
Fear, anxiety	.580**	.587**	.459**	.500**
**School results**				
Mathematics average	-.460**	-.437**	-.327**	-.486**
French average	-.273**	-.266**	-.197**	-.273**
**Perceived stress**				
Total perceived stress	.712**	.684**	.605**	.614**
Stress related to success	.683**	.657**	.540**	.639**
Stress related to relationships	.527**	.529**	.439**	.431**
Stress related to workload	.628**	.578**	.562**	.538**
**Interpersonal factors**				
Support from teachers	-.460**	-.414**	-.444**	-.366**
Support from parents	-.439**	-.364**	-.440**	-.370**
Support from friends	-.413**	-.359**	-.368**	-.385**
Support from peers	-.522**	-.498**	-.478**	-.411**
**Covid-19 related factors**				
Increased workload after COVID-19	.402**	.373**	.352**	.349**
Getting back to life before COVID-19	-.495**	-.435**	-.437**	-.460**
Adequacy of current support	-.344**	-.271**	-.334**	-.324**
Adequacy of support during containment	-.180*	-.127 (ns)	-.180*	-.182*
Trust in future	-.551**	-.501**	-.469**	-.512**

*Note. Correlation is significant at the **p < 0.01; BO = Burnout ; EE = Exhaustion; CYN = Cynicism ; INAD = Inadequacy*

Table S2 : Results of ANOVA between burnout and organizational factors

	BO		EE		CYN		INAD	
	M	*SD*	*M*	*SD*	*M*	*SD*	*M*	*SD*
Nombre d'enseignants								
1 enseignant (*n*= 7)	2.44	.85	2.29	1.11	2.43	.74	2.79	1.58
2 enseignants (*n*= 77)	2.19	1.06	1.95	.92	2.33	1.24	2.45	1.46
3 enseignants (*n*= 48)	2.38	1.01	2.14	1.03	2.42	1.22	2.82	1.39
4 enseignants ou plus (*n*= 52)	2.47	1.09	2.28	1.05	2.47	1.30	2.85	1.41
Nombre d'élèves								
Moins de 14 (*n*= 12)	2.13	1.02	1.88	.68	2.22	1.47	2.50	1.26
Entre 14 et 17 (*n*= 31)	2.43	.91	2.15	.82	2.61	1.10	2.74	1.52
Entre 18 et 21 (*n*= 97)	2.42	1.03	2.19	1.03	2.45	1.21	2.85	1.38
Entre 22 et 25 (*n*= 44)	2.11	1.17	1.97	1.10	2.17	1.30	2.30	1.47
